# Supplementary material for: Photoexcitation of Ge9− Clusters in THF: New Insights into the Ultrafast Relaxation Dynamics and the Influence of the Cation
Source: Molecules. 2020 Jun 5;25(11):2639. doi: 10.3390/molecules25112639 (PMC7321105; doi:10.3390/molecules25112639)
Supplement: Supplementary file 1 [file molecules-25-02639-s001.pdf]

Supplementary Information

Photoexcitation of Ge_9^- Clusters in THF: New Insights into the Ultrafast Relaxation Dynamics and the Influence of the Cation

N. C. Michenfelder,[1] C. Gienger,[2] M. Dilanas,[1] A. Schnepf[2]* and A.-N. Unterreiner[1]*

[1]Institut für Physikalische Chemie, Karlsruher Institut für Technologie (KIT), Kaiserstr. 12, 76131 Karlsruhe, Germany
[2]Institut für Anorganische Chemie, Universität Tübingen, Auf der Morgenstelle 18, 72076 Tübingen, Germany

* A.-N. Unterreiner. E-Mail: andreas.unterreiner@kit.edu. Tel: +49 721 608 47807.
* A. Schnepf. Email: andreas.schnepf@uni-tuebingen.de. Tel.: +49 7071 29 76635. Fax: +49 (7071) 28 – 2436.

Stationary absorption spectra

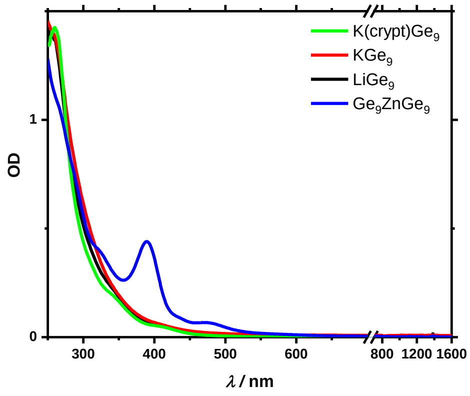

Figure S 1. Absorption spectra of all investigated compounds K(crypt)Ge_9, KGe_9, $LiGe_9$ and Ge_9ZnGe_9 in THF between 250 and 1600 nm.

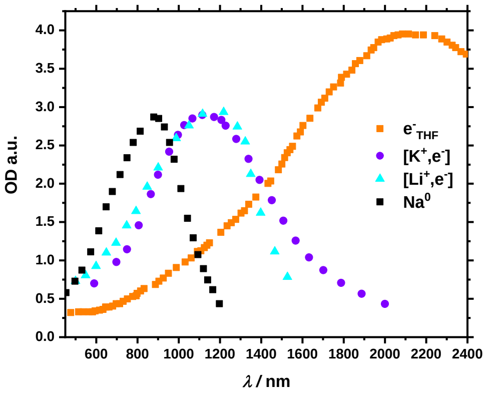

Figure S 2. As reference for the analysis to illustrate the NIR spectra digitized absorption spectra of the solvated electron (e^-_{THF}), the $[K^+,e^-]$ contact pair and the $[Li^+,e^-]$ contact pair and Na^0 in THF between 450 and 2400 nm from references [37], [40], [39] and [16], respectively.

Transient absorption spectra

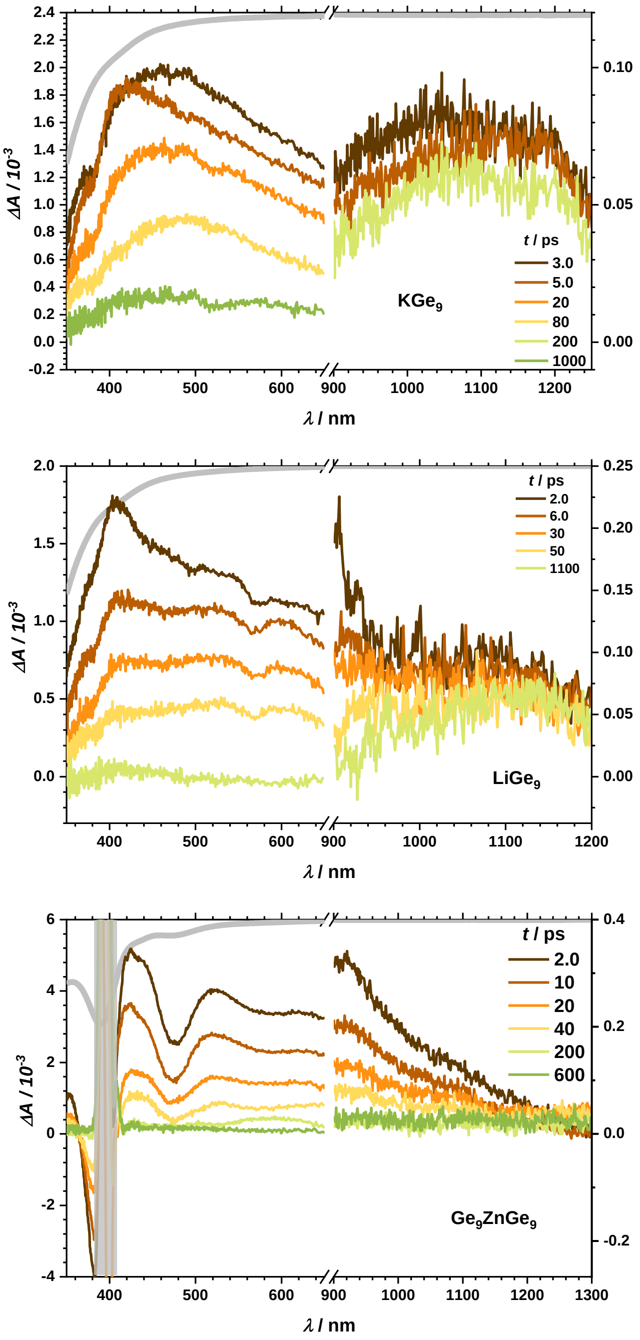

Figure S 3. Additional transient absorption spectra in the UV-Vis and NIR spectral range at selected delay times after 267/258 nm excitation of KGe_9 (top), $LiGe_9$ (middle) ($K(crypt)Ge_9$ and Ge_9ZnGe_9 can be found in the main manuscript) and after 400/388 nm excitation of Ge_9ZnGe_9 (bottom) in THF. After 400 nm excitation scattered light of the pump pulse superimposes the spectra marked as gray area. KGe_9 and $LiGe_9$ do not differ much from $K(crypt)Ge_9$ but the long lived absorption seems to have a maximum around 1050-1100 nm. For Ge_9ZnGe_9 the intensities are higher compare to the monomers and the transient response is shorter especially after 400/388 nm excitation.

Transient absorption traces

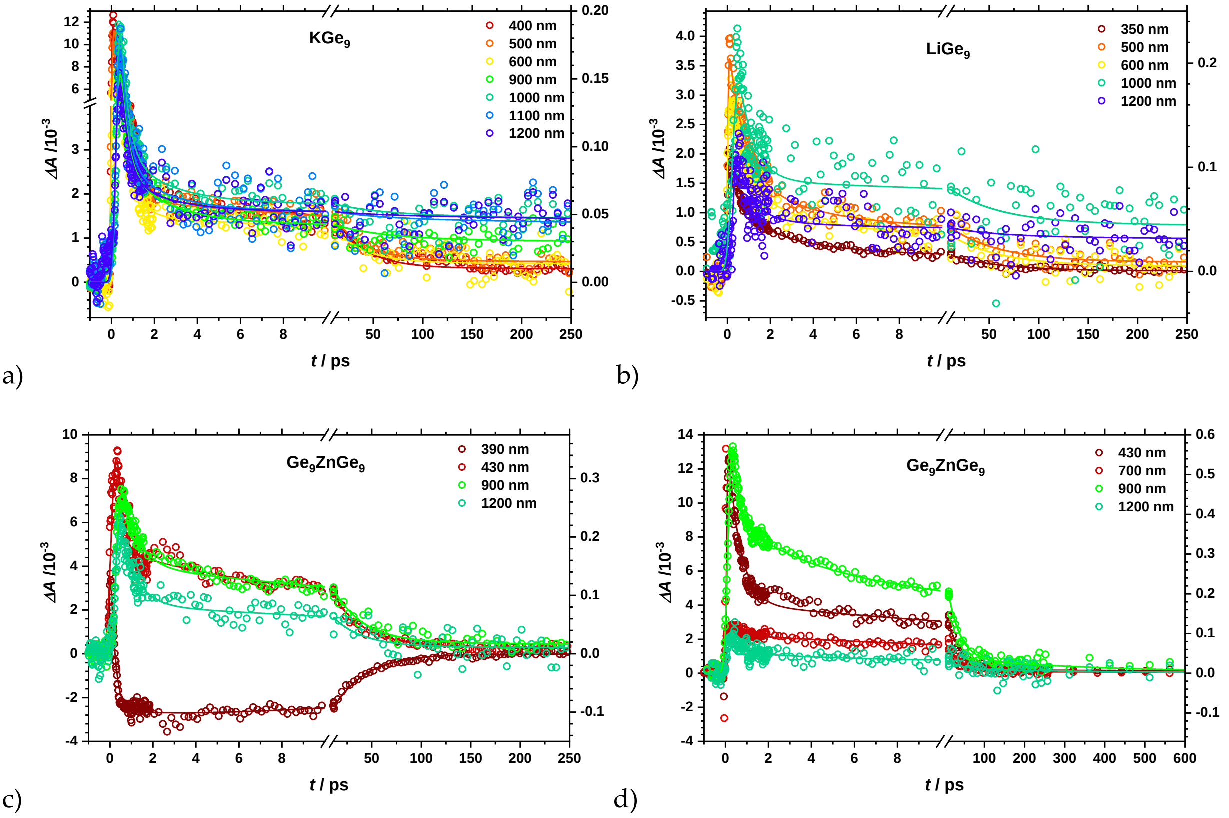

Figure S 4. Additional transient response at different probe wavelengths (circles) for KGe_9 (a)), $LiGe_9$ (b)) and Ge_9ZnGe_9 (c)) after 267/258 nm excitation and the latter after 400/388 nm (d)) in THF. Note the different intensity scales for UV-Vis (left axis) vs. NIR (right axis) probe wavelengths. Fit curves are displayed as lines. Due to different techniques, the time resolution of the UV-Vis excited experiments is considerably shorter than in the NIR region as obvious by a faster rise of the corresponding transients on an ultrashort timescale. The long timescale dynamics is pronounced in the NIR spectral region. For Ge_9ZnGe_9 the ground state bleach leads to a negative transient response at 390 nm the NIR intensity ratios only differ slightly from the ones in the visible region.

Decay associated spectra

Fit function:

$$\Delta A(t, \lambda_{probe}) = \frac{1}{2}\left(1 + erf\left[\sqrt{4 \cdot ln(2)}\frac{t}{\tau_0}\right]\right) \cdot \left[\sum_{i=1}^{n} A_i(\lambda_{probe}) \cdot e^{-\frac{t}{\tau_i}}\right]$$

Global analysis was done with an in-house written program in Matlab environment. The transients were fitted to a model function consisting in the first term of an error function to fit the first rise after photoexcitation. As gauss-functions are assumed for the pump and probe pulses, the deconvolution is mathematically approximated with an error function. In the second term n (in this case four) exponential decay components each with identical time constants τ_i through all over all wavelengths in the UV-Vis and NIR regions separately were used to fit the evolution of the excited state population. The four time constants from the fitting procedure are given in Table 1 in the main manuscript and the corresponding amplitudes A_i are presented as decay associated spectra (DAS). The DAS show the absorption spectra of the excited states, which correspond to the respective time constants or decay processes.

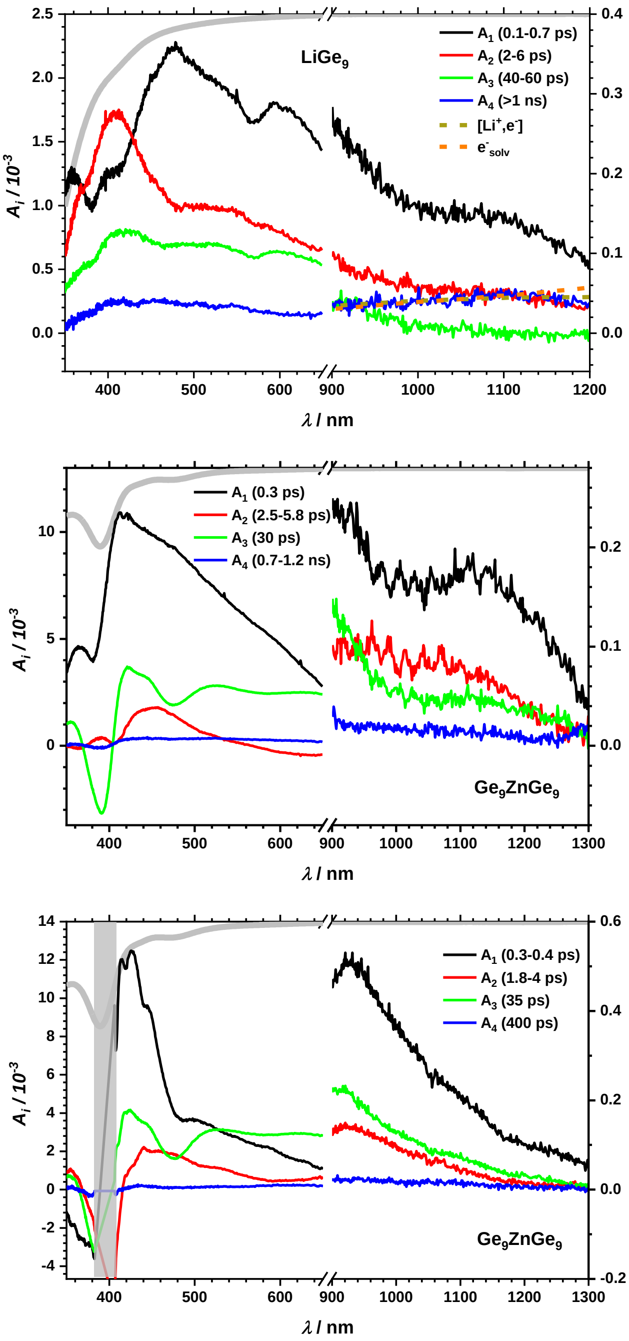

Figure S 5. Additional decay associated spectra from global fit for $LiGe_9$ (top) Ge_9ZnGe_9 after 267/258 nm (middle) and 400/388 nm (bottom) excitation. UV-Vis and NIR spectra were fitted separately. Discrepancies in time constants are given in ranges. After 400 nm excitation scattered light of the pump pulse superimposes the spectra marked as gray area. For $LiGe_9$ the spectrum of $[Li^+,e^-]$ is depicted in dark yellow color and of the solvated electron in THF in orange. The $[Li^+,e^-]$ fits the A_4 spectrum in this region. For Ge_9ZnGe_9 no match for a cation electron contact pair or a solvated electron spectrum could be found.

X-Ray Powder Diffraction

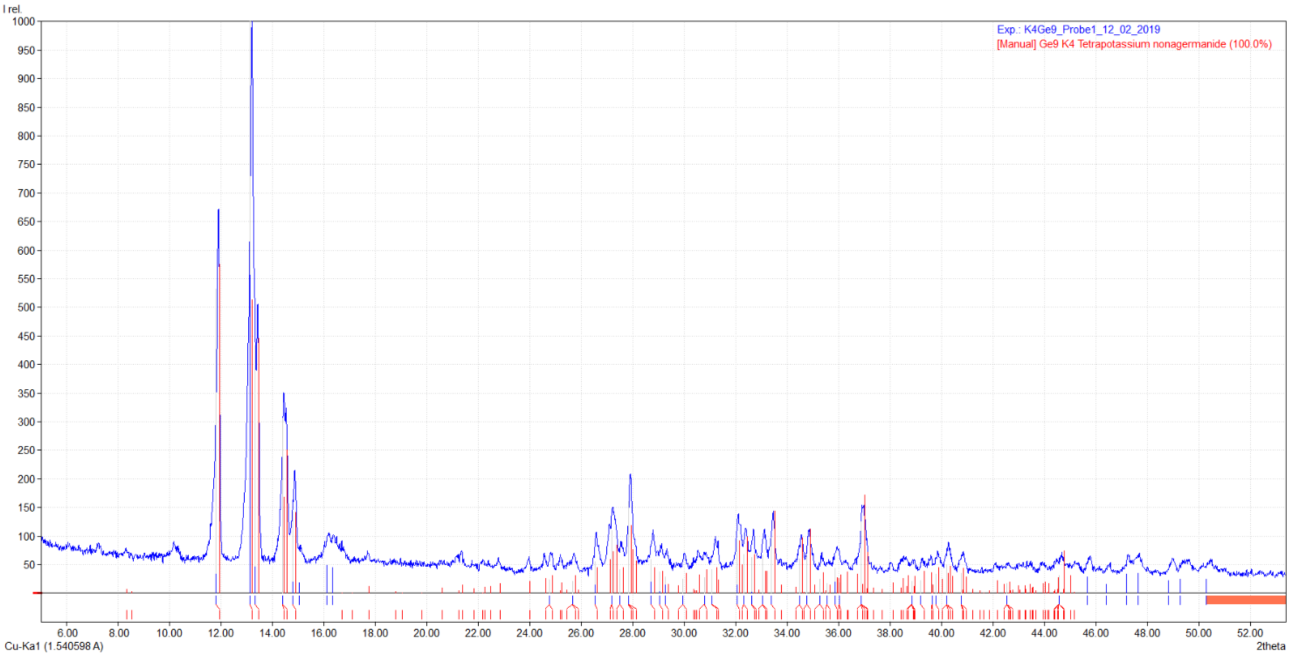

Figure S 6: PXRD pattern of K_4Ge_9 (blue) with simulated pattern from single crystals (red) underneath. The broad reflection around 16° originates from Leybonol® that was used to prepare the sample for measurement.
